# Comparison of air displacement plethysmography to hydrostatic weighing for estimating total body density in children

**DOI:** 10.1186/1471-2431-5-37

**Published:** 2005-09-09

**Authors:** Geo Claros, Holly R Hull, David A Fields

**Affiliations:** 1 Department of Health and Exercise Science, University of Oklahoma, Norman, OK, USA; 2 Department of Pediatrics, University of Oklahoma Health Science Center, Oklahoma City, OK, USA; 3 Children's Medical Research Institute's Metabolic Research Center, University of Oklahoma Health Science Center, OUCP Diabetes and Endocrinology, Oklahoma City, OK, USA

## Abstract

**Background:**

The purpose of this study was to examine the accuracy of total body density and percent body fat (% fat) using air displacement plethysmography (ADP) and hydrostatic weighing (HW) in children.

**Methods:**

Sixty-six male and female subjects (40 males: 12.4 ± 1.3 yrs, 47.4 ± 14.8 kg, 155.4 ± 11.9 cm, 19.3 ± 4.1 kg/m^2^; 26 females: 12.0 ± 1.9 yrs, 41.4 ± 7.7 kg, 152.1 ± 8.9 cm, 17.7 ± 1.7 kg/m^2^) were tested using ADP and HW with ADP always preceding HW. Accuracy, precision, and bias were examined in ADP with HW serving as the criterion method. Lohman's equations that are child specific for age and gender were used to convert body density to % fat. Regression analysis determined the accuracy of ADP and potential bias between ADP and HW using Bland-Altman analysis.

**Results:**

For the entire group (Y = 0.835x + 0.171, R^2 ^= 0.84, SEE = 0.007 g/cm^3^) and for the males (Y = 0.837x + 0.174, R^2 ^= 0.90, SEE = 0.006 g/cm^3^) the regression between total body density by HW and by ADP significantly deviated from the line of identity. However in females, the regression between total body density by HW and ADP did not significantly deviate from the line of identity (Y = 0.750x + 0.258, R^2 ^= 0.55, SEE = 0.008 g/cm^3^). The regression between % fat by HW and ADP for the group (Y = 0.84x + 3.81, R^2 ^= 0.83, SEE = 3.35 % fat) and for the males (Y = 0.84x + 3.25, R^2 ^= 0.90, SEE = 3.00 % fat) significantly deviated from the line of identity. However, in females the regression between % fat by HW and ADP did not significantly deviate from the line of identity (Y = 0.81x + 5.17, R^2 ^= 0.56, SEE = 3.80 % fat). Bland-Altman analysis revealed no bias between HW total body density and ADP total body density for the entire group (R = 0.-22; *P *= 0.08) or for females (R = 0.02; *P *= 0.92), however bias existed in males (R = -0.37; *P *≤ 0.05). Bland-Altman analysis revealed no bias between HW and ADP % fat for the entire group (R = 0.21; *P *= 0.10) or in females (R = 0.10; *P *= 0.57), however bias was indicated for males by a significant correlation (R = 0.36; *P *≤ 0.05), with ADP underestimating % fat at lower fat values and overestimating at the higher % fat values.

**Conclusion:**

A significant difference in total body density and % fat was observed between ADP and HW in children 10–15 years old with a potential gender difference being detected. Upon further investigation it was revealed that the study was inadequately powered, thus we recommend that larger studies that are appropriately powered be conducted to better understand this potential gender difference.

## Background

Recent results obtained from the National Health and Nutrition Examination Survey (NHANES) found increases in overweight and obesity not only in adults but also in children [1]. An alarming 31% of children aged 6 to 19 years old were found to be at risk for overweight while 16% were classified as overweight. At this time, there is no indication of this trend abating but in fact only growing worse. These facts stress the importance of accurate methods to determine body composition in a pediatric population to identify children at risk of becoming obese or those who are already obese.

Several methods can be used to determine body composition in a pediatric population, however these techniques can be costly, time consuming and difficult to administer in a pediatric population. Hydrostatic weighing (HW) has commonly been used in adults, though the administration in certain populations such as the elderly, ill, children, and certain ethnic groups has proven challenging. For successful completion of HW, multiple trials of complete head submersion followed by a maximal exhalation to record underwater weight is the likely culprit for this difficulty. In our study alone 9 children were unable to achieve the requirements necessary to complete a HW test.

Air displacement plethysmography (ADP) is a method that offers promise to alleviate problems associated with HW and is widely used to assess body composition of adults in many different settings. An ADP test involves the child sitting inside of the testing chamber while wearing a swimsuit, swim cap and nose clips for assessment of body volume with each body volume measurement lasting only 50 seconds [2]. After two successful body volume measurements, thoracic lung volume is measured using a tube placed inside of the testing chamber and using the gentle puffing maneuver. Total time to assess body composition by ADP in children is approximately 8–10 minutes compared to upwards of 30 minutes or more for HW.

Several studies have investigated the validity and feasibility of ADP in an adult population and found ADP to be valid and reliable [3-9], while others have not found agreement [10]. Although numerous studies have been completed in an adult population, a limited number of studies exist in evaluating ADP in a pediatric population [6,9,11-15] with two studies measuring the residual lung volume simultaneously [13,14].

Therefore, the purpose of this study was to evaluate body density obtained by ADP to body density obtained by HW in children and adolescents and to examine if gender differences exist between ADP and HW estimates in body density and % fat.

## Methods

### Subjects

A total of 77 subjects were recruited through the Norman Youth Soccer Association. Norman, Oklahoma. Two subjects' data were invalid due to instrument malfunction while an additional 9 subjects were unable to perform the HW procedures. Therefore, data from only 66 subjects were used in the analysis, of which 40 were males and 26 females between the ages of 10 – 15 years old. Subjects were excluded from participation in the study if they were claustrophobic or had any known lung disease or disorder, including asthma.

### Protocol

Subjects were required to visit the Human Body Composition Laboratory at the University of Oklahoma Norman Campus for one visit with the visit lasting approximately 1 1/2 hours. ADP always preceded HW, this was done to ameliorate any residual effect that heat and moisture may have on ADP measurements [16]. Written informed consent and assent were obtained from all subjects and their parents. This research study was approved by the University of Oklahoma Institutional Review Board.

### BOD POD instrumentation

The BOD POD^® ^Body Composition System (Life Measurement Instruments, Concord, CA) was used to assess body volume and total body density with the operating procedures previously described elsewhere [2]. Total body density was converted to percent fat using the Lohman age specific equations [17]. Subjects were instructed not to eat, drink or exercise 6 hours prior to testing. All subjects wore a Speedo swimsuit (provided by the laboratory), swim cap, nose clips, and removed all jewelry prior to testing. The traditional and well established pulmonary plethysmographic measurement (used inbasic pulmonary function testing) of thoracic gas volume (TGV) is adopted by the BOD POD and incorporated into the testingfor TGV measurement and has been shown to be valid [2,18]. The only difference is that the traditional pulmonary plethysmography determines TGV at the end-tidal exhalation; however, the BOD PDO measures TGV at mid-tidal exhalation. This is done because it is necessary to correct raw body volume for the average amount of airin thelungs during normal tidal breathing, which is reflected by taking the measurement at mid-tidal exhalation. The testing procedure involved the following steps. First, the BOD POD was calibrated by computing the ratio of the pressure amplitudes (reference chamber and testing chamber) for the empty testing chamber, which is ~ 450L, and the testing chamber with our calibration cylinder (49.556-L). Following the calibration and after the TGV procedure was explained and all pertinent subject data entered into the computer software, the subject entered the testing chamber to have there raw body volume measured. After body volume was measured, the TGV was measured. The TGV was measured by having the subject sit quietly in the BOD POD while breathing through a disposable tube and filter connected to the reference chamber in the rear of the BOD POD. After four or five normal breaths and at the point of mid-exhalation the airway was occluded and the subject was instructed to make two quick and light puffs. All TGV measurements were measured and not estimated.

### Hydrostatic weighing

Each child's total body density was measured by underwater weighing, with a simultaneous measurement of residual lung volume by using the closed-circuit oxygen dilution technique measurement system (EXERTECH, Dresbach, MN). The simultaneous residual lung volume measurement system included a calibrated piston pump which was used to dispense a measured volume of oxygen into a rubber bag and a fast responding electronic nitrogen gas analyzer. This continuously sampled the inhaled and exhaled gas at the subject's mouth in order to follow the nitrogen fraction of the respiratory air as it mixed with the pre-measured oxygen bag volume during re-breathing (i.e. when the subjects emerged from being submerged under the water). The nitrogen gas fraction of the mixture was continuously recorded during the re-breathing procedure and reached a relative equilibrium, usually within 5 or 6 breaths. Residual volume was calculated from the initial oxygen volume in the bag and the change in nitrogen fraction by dilution. The underwater weight was measured to the nearest 1/100^th ^of a gram in an enclosed tile tank in which the subject, while wearing a one-piece swimsuit, sat in a carriage wrack suspended from four LCL 10 load cells integrated with a summing box and digital display calibrated from 0 to 18,000 g (Omega, Stanford, CT). After two practice trials, underwater weight and the residual lung volume were measured simultaneously 5 times. The average of multiple trial densities within 1/1000^th ^g/cm^3 ^were used for the underwater weight. Percent fat mass was calculated from whole body density (g/cm^3^) using Lohman age specific equations [17].

### Data analysis

Accuracy, precision, and bias were examined in ADP with HW serving as the criterion method. Statistical significance was set at (*P *≤ 0.05).

Regression analysis was used to determine the accuracy of ADP. ADP was considered to be accurate if the regression between body density and percent fat by HW and ADP did not have a slope significantly different from one and an intercept significantly different from zero. This analysis tested the hypothesis that the regression of body density and percent fat by HW and body density and percent fat by ADP did not significantly deviate from the line of identity.

The amount of shared variance between ADP and HW was assessed by R^2^. Potential bias between ADP and HW were examined using Bland-Altman analysis [19]. This particular test examined the difference in body density and percent fat between ADP and HW as a function of the average body density and percent fat by ADP and HW. A non-significant correlation indicated no bias was seen in the technique (i.e. ADP) across the range of fatness.

## Results

**Figure 1:**
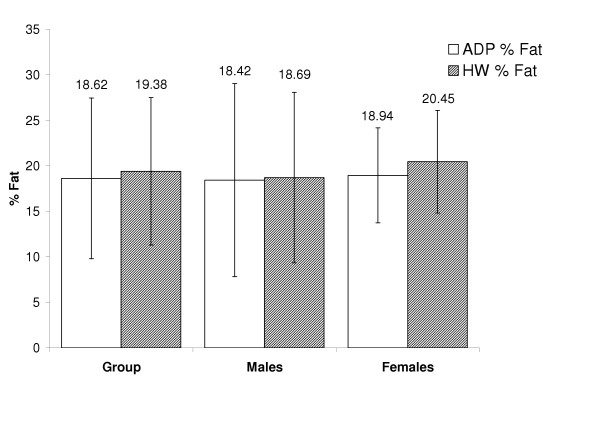
The bars represent % fat measurements by ADP and HW for the entire group and for males and females.

The purpose of this study was to compare ADP with HW in male and female children and adolescents between the ages of 10–15 years old. Results for this study will be presented in the following order; body density findings followed by % fat findings. The physical characteristics of the subjects are presented in Table 1 and a summary of % fat means for the total group and for each gender are found in Figure 1.

**Table 1:** Physical Characteristics of subjects

	Group (*N *= 66)	Males (*N *= 40)	Females (*N *= 26)
Age, (yr)	12.2 ± 1.6	12.4 ± 1.3	12.0 ± 1.9
Body weight, (kg)	45.0 ± 12.8	47.4 ± 14.8	41.4 ± 7.7
Height, (in)	60.7 ± 4.3	61.2 ± 4.7	59.9 ± 3.5
BMI (kg/m^2^)	18.7 ± 3.4	19.3 ± 4.1	17.7 ± 1.7
ADP body density (g/cm^3^)	1.048 ± 0.019	1.050 ± 0.023	1.045 ± 0.011
HW body density (g/cm^3^)	1.046 ± 0.017	1.049 ± 0.020	1.042 ± 0.011
ADP % fat	18.6 ± 8.8	18.4 ± 10.6	18.9 ± 5.2
HW % fat	19.4 ± 8.1	18.7 ± 9.4	20.4 ± 5.6

### Total body density

**Figure 2:**
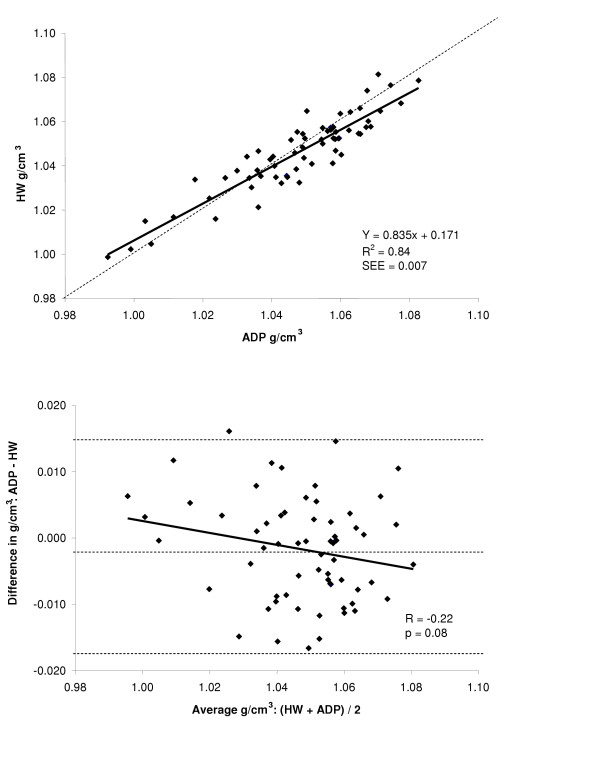
*Top*: regression of body density (g/cm^3^) by ADP against body density by HW for the total group. *Bottom*: Bland-Altman analysis for the group where the middle dashed line represents the mean difference between body density by ADP – body density by HW. The upper and lower dashed lines represents ± 2 SD from the mean. No bias between the techniques was observed as indicated by a nonsignificant *P *value.

**Figure 3:**
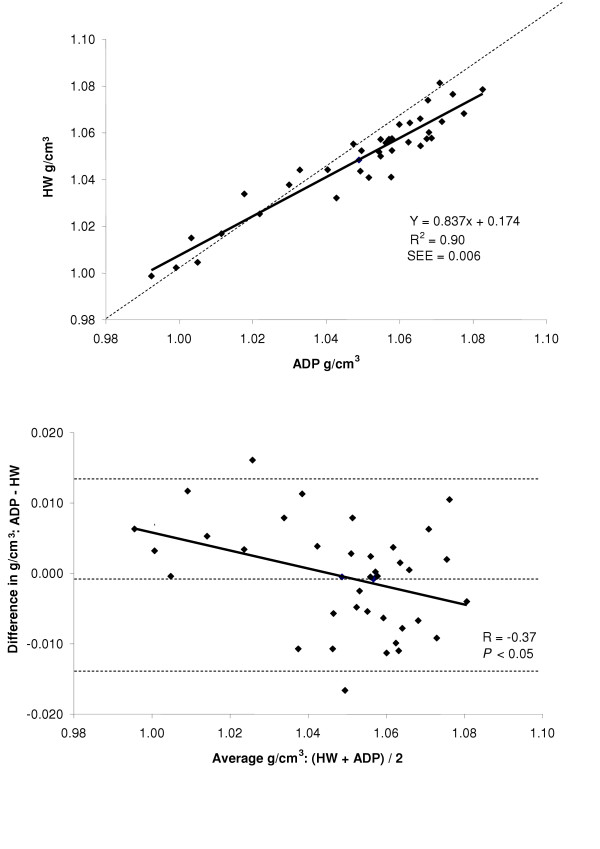
*Top*: regression of body density (g/cm^3^) by ADP against body density by HW for males. *Bottom*: Bland-Altman analysis for the group where the middle dashed line represents the mean difference between body density by ADP – body density by HW. The upper and lower dashed lines represents ± 2 SD from the mean. Bias between the techniques was observed as indicated by a significant *P *value.

**Figure 4:**
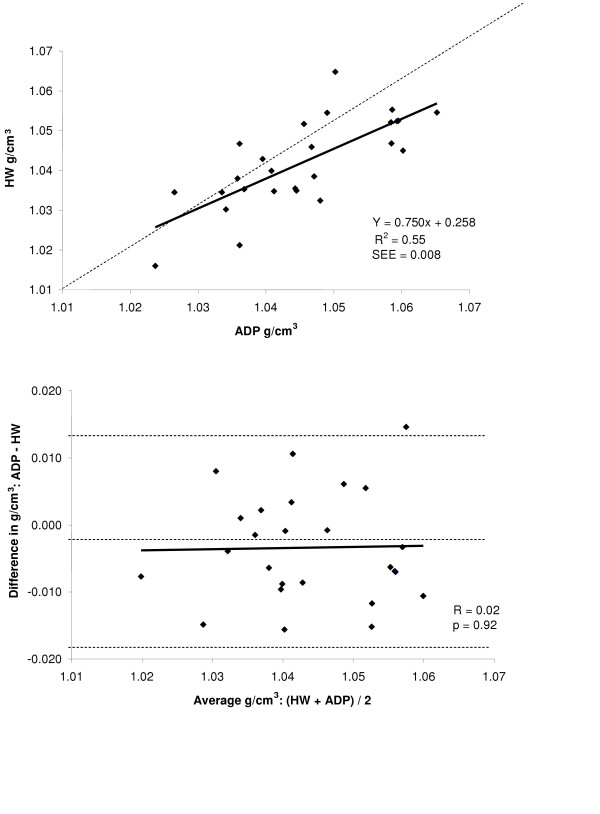
*Top*: regression of body density (g/cm^3^) by ADP against body density by HW for females. *Bottom*: Bland-Altman analysis for the group where the middle dashed line represents the mean difference between body density by ADP – body density by HW. The upper and lower dashed lines represents ± 2 SD from the mean. No bias between the techniques was observed as indicated by a nonsignificant *P *value.

Accuracy and the amount of shared variance between the techniques of body density was examined by regression of HW body density versus body density by ADP for the total group and for each gender. The regressions between the two methods are shown in the top panel of Figures 2, 3, and 4 for the group, males, and females respectively, while a summary of these regressions are presented in Table 2. The group (Y = 0.835x + 0.171, R^2 ^= 0.84, SEE = 0.007 g/cm^3^) and male regressions (Y = 0.837x + 0.174, R^2 ^= 0.90, SEE = 0.006 g/cm^3^) of ADP total body density compared to HW total body density significantly deviated from the line of identity. However, for the females the regression between ADP total body density and HW total body density did not significantly deviate from the line of identity (Y = 0750x + 0.258, R^2 ^= 0.55, SEE = 0.008 g/cm^3^).

**Table 2:** Summary of regression for density

	R^2^	Intercept, (g/cm^3^)	Slope	SEE (g/cm^3^)
ADP group	0.84	0.171 ± 0.049*	0.835 ± 0.046**	0.007
ADP males	0.90	0.171 ± 0.048*	0.837 ± 0.045**	0.007
ADP females	0.55	0.258 ± 0.146	0.750 ± 0.140	0.008

*Significantly different from 0 (*P *< 0.05)

**Significantly different from 1 (*P *< 0.05)

A Bland-Altman analysis was performed to determine whether bias existed between ADP and HW total body density across the range of fatness for the entire group and for each gender. These analyses are presented in bottom panel of Figures 2, 3, and 4 for the entire group, males, and females respectively. For the group (R = -0.22; *P *= 0.08) and for females (R = 0.02; *P *= 0.92), no bias was observed as indicated by a non significant correlation. However, in males bias existed across the range of fatness (R = -0.37; *P *≤ 0.05), with ADP overestimating total body density at lower densities and underestimating total body density at higher densities.

### % Fat

**Figure 5:**
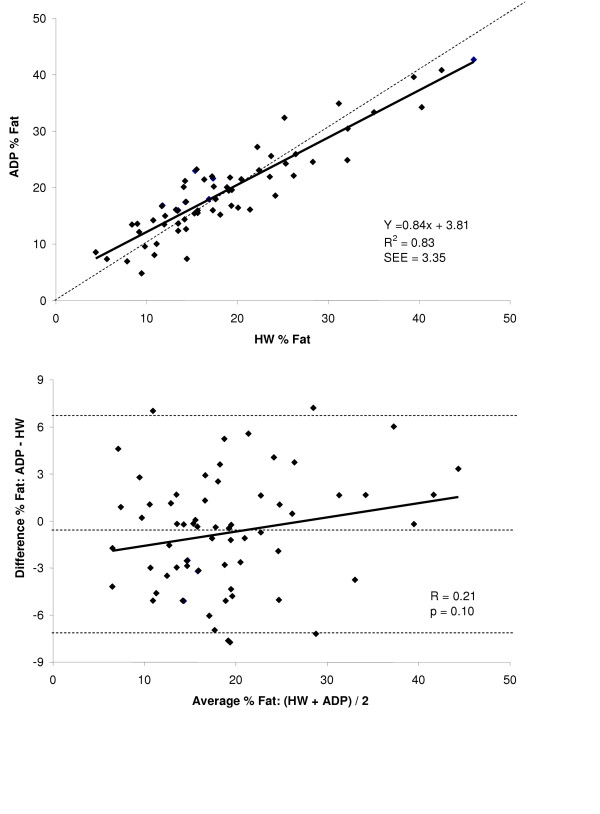
*Top*: regression of % fat by ADP against % fat by HW for the total group. *Bottom*: Bland-Altman analysis for the group where the middle dashed line represents the mean difference between % fat by ADP – % fat by HW. The upper and lower dashed lines represents ± 2 SD from the mean. No bias between the techniques was observed as indicated by a nonsignificant *P *value.

**Figure 6:**
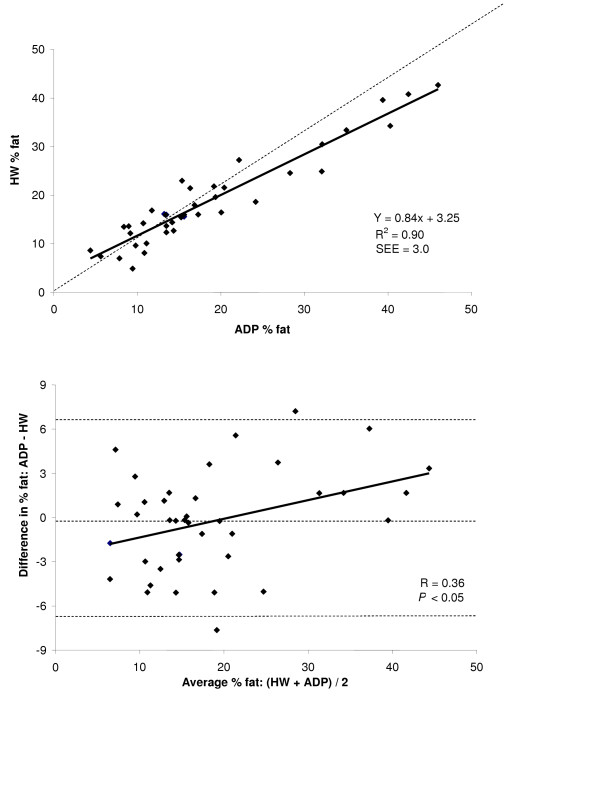
*Top*: regression of % fat by ADP against % fat by HW for males. *Bottom*: Bland-Altman analysis for the group where the middle dashed line represents the mean difference between % fat by ADP – % fat by HW. The upper and lower dashed lines represents ± 2 SD from the mean. Bias between the techniques was observed as indicated by a significant *P *value.

**Figure 7:**
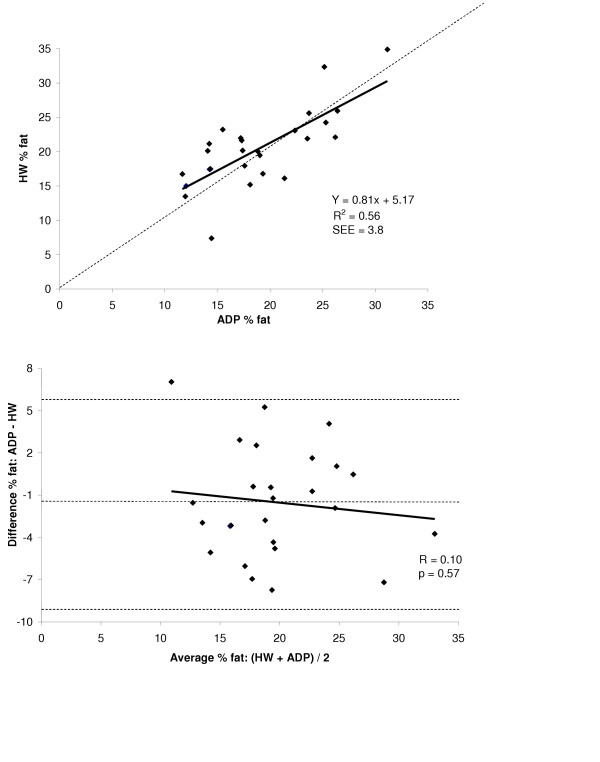
*Top*: regression of % fat by ADP against % fat by HW for females. *Bottom*: Bland-Altman analysis for the group where the middle dashed line represents the mean difference between % fat by ADP – % fat by HW. The upper and lower dashed lines represents ± 2 SD from the mean. No bias between the techniques was observed as indicated by a nonsignificant *P *value.

A summary of % fat estimates for the entire group and for each gender for both HW and ADP are shown in Figure 1 and the summary of the regressions for % fat for the group and both genders are shown in Table 3. Accuracy of % fat was examined by the regression of HW % fat against ADP % fat for the total group and for each gender. These regressions are shown in the top panel Figures 5, 6, and 7 for the group, males, and females respectively. The regression between % fat by HW and % fat by ADP significantly deviated from the line of identity for the entire group (Y = 0.84x + 3.81, R^2 ^= 0.83, SEE = 3.35 % fat) and in the males (Y = 0.84x + 3.25, R^2 ^= 0.90, SEE = 3.00 % fat). However, in females the regression did not significantly deviated from the line of identity (Y = 0.81x + 5.17, R^2 ^= 0.56, SEE = 3.80 % fat).

**Table 3:** Summary of regression for % fat

	R^2^	Intercept, (kg)	Slope	SEE (% fat)
ADP group	0.83	3.81 ± 0.97*	0.84 ± 0.05**	3.4
ADP males	0.90	3.25 ± 0.96*	0.84 ± 0.05**	3.0
ADP females	0.56	5.17 ± 2.88	0.81 ± 0.15	3.8

*Significantly different from 0 (*P *< 0.05)

**Significantly different from 1 (*P *< 0.05)

A Bland-Altman analysis was performed to determine whether bias existed between ADP and HW across the range of body fatness for the group and for each gender. These analyses are shown in bottom panel of Figures 5, 6, and 7 for the group, males, and females respectively. ADP for the group (R = 0.21; *P *= 0.10) and for females (R = 0.10; *P *= 0.57) did not show a significant bias across the range of body fatness. ADP for males showed a significant bias across the range of body fatness (R = 0.36; *P *≤ 0.05), with ADP underestimating body fat at lower fat values and overestimating at the higher fat values.

### Lung volumes

A paired t – test was conducted to determine if the measured lung volume (e.g. residual lung volume in hydrostatic weighing and the thoracic gas volume in ADP) significantly deviated from its respective estimated lung volumes. A summary of the lung volumes are presented in Table 4. A significant over-estimation in the residual lung volume was observed between the measured and predicted residual lung volume for the group (1.27 ± 0.44 vs. 0.84 ± 0.12) and for both males (1.36 ± 0.48 vs. 0.86 ± 0.14) and females (1.12 ± 0.33 vs. 0.82 ± 0.10), as indicated by a *P *≤ 0.05. A significant under-estimation in the thoracic gas volume was also observed between the measured and predicted thoracic gas volume, volume for the group (2.45 ± 0.68 vs. 2.71 ± 0.51) for the males, (2.53 ± 0.72 vs. 2.74 ± 0.58) and for the females (2.33 ± 0.62 vs. 2.66 ± 0.36) indicated by P ≤ 0.05.

**Table 4:** Summary of residual volume and thoracic gas volumes for the total group and for both genders

	Residual Volume (predicted^1^)	Residual Volume (measured)	TGV (predicted^2^)	TGV (measured)
Group	0.84 ± 0.12	1.27 ± 0.44**	2.71 ± 0.51	2.45 ± 0.68**
Males	0.86 ± 0.14	1.36 ± 0.48*	2.74 ± 0.58	2.53 ± 0.72*
Females	0.82 ± 0.10	1.12 ± 0.33*	2.66 ± 0.36	2.33 ± 0.62*

* Statistically significant from predicted (*P *< 0.05)

** Statistically significant from predicted (*P *< 0.01)

^1 ^Polgar, 1971 RV = (0.029 × Ht(in)) - 0.919

^2 ^Crapo, 1982 VTG = FRC + 1/2 TV

## Discussion

The purpose of this study was to validate ADP with HW in a group of children of varying degrees of fatness. This study is significant because the ability to accurately measure body composition in children is challenging and difficult. With the sudden increase in the incidence of pediatric obesity, the ability to accurately determine body composition is paramount in the treatment of this escalating problem. ADP has shown to be a valid tool in an adult population [3-9,20] however, few studies have compared ADP and HW in children [6,11-15]. Thus, the purpose of this study was to compare total body density and % fat measurements obtained by ADP against those obtained by HW in male and female children between the ages of 10–15 years old.

Prior studies validating ADP and HW in children have produced varying results. Lockner et al. studied 54 children and found agreement between the two techniques by regression analysis [11]. Although regression indicated agreement between techniques, a significant group mean difference was found (*P*<0.0005). Dewit et al. also found no difference between total body density by HW and total body density by ADP in children ranging in age from 8–12 years old [14]. Further research comparing in children has found differences between ADP and HW. Nunez et al. studied a large sample of children consisting of 54 males and 66 females [6]. Regression analysis revealed poor agreement between total body density by HW and total body density by ADP for the entire group of children. Further analysis by Nunez et al. separated % fat findings by gender and found a significant gender difference. Percent fat measured by ADP was significantly greater than % fat measured by HW in both females and males by 1.7% and 0.5%, respectively (*P *< 0.0001).

Possible reasons for disagreement in the literature found between ADP and HW in children are numerous. When assessing body composition by ADP some studies predicted TGV instead of actually measuring TGV [6,11]. Research has shown using the prediction equations originally developed for adults in children will overestimate TGV resulting in inaccurate % fat measures [21]. Traditionally, measuring TGV has proven challenging in a pediatric population with 35% of pediatric centers unable to measure the TGV. It should be noted, all of the TGV measurements in this study were measured, and none were predicted. Some studies did not follow strict protocol and allowed subjects to use clothing other than a tight fitting swimsuit such as spandex bicycle shorts [6,11,22]. When a strict clothing protocol is not followed, it has been shown that % fat can be underestimated upwards of 6% [23,24]. When converting body density to % fat, the correct child specific equation must be used. Both Demerath et al. and Lockner et al. used the Siri equation to convert body density to % fat instead of the child gender specific Lohman equations [11,12]. And lastly, in a study by Lockner et al. the testing order was randomized, thus some of the children performed an ADP after performing a HW [11]. It has been shown by Fields et al., that HW prior to ADP resulted in an underestimation of % fat by approximately 3% [16].

One of the purposes of this study was to examine potential gender differences between ADP and HW. The results from this study did show significant gender difference between techniques, with males significantly deviating from the line of identity. However, this was not found in females for either total density or % fat. Nonetheless, this gender difference reported in this study should be viewed with caution because a power analysis revealed that 40 subjects per gender (Group N = 80) were needed for 80% power (alpha = 0.05) for a main effect of 1.5% fat. In this study only 26 females were used in the data analysis, though 37 were brought in for testing. This was due to the fact that 9 of the female subjects were unable to perform the HW procedures and two were lost when equipment malfunctioned. A power analysis for this data set was performed and it was found after factoring in those dropped from the study, there remained only a power of 0.429. Therefore, we did not have enough power to detect the potential difference between the methods in females.

Research has shown for ADP that prediction equations developed in adults provide invalid lung volume predictions in children. The adult prediction equations used to predict TGV were developed using a healthy adult population [25]. Fields et al. measured TGV in 113 boys and 111 girls and found prediction equations significantly overestimated TGV in both genders (*P *< 0.001) [21]. In the current study, residual lung volume was measured simultaneously in water and TGV was measured during testing. This is significant because only two other studies have been able to simultaneously measured residual lung volume while underwater weighing in children [13,14].

Interestingly, the coefficient of variance (CV) for repeated measures over two days in a subset of the children in this study for ADP and HW was 3.1% and 7.1% respectively. This is quite high considering the CV for ADP and HW in our laboratory for adults are 1% and 1.5% respectively, though Nunez et al. reported an ADP CV of 8.5% in a pediatric population [6]. Consequently, the high CV may be playing a role in the true relationship between ADP and HW.

## Conclusion

Due to the ease in the testing procedure and high subject compliance for all ages, ADP has quickly begun to emerge as a popular body composition method to use in children and adults. In the current study, we found an overall poor agreement between ADP and HW with the study inadequately powered to make any definitive statements concerning potential gender differences between the techniques. In conclusion, we recommend more studies validating ADP and HW in children be performed utilizing a larger sample size.

## Competing interests

David A. Fields has received funding from Life Measurement Incorporated for past studies (though Life Measurement Incorporated did not fund this study). None of the authors have non-financial competing interests or own stock or are applying for patents that may represent a conflict of interest.

## Authors' contributions

GC carried out the hydrostatic weighing and ADP studies, participated in the design of the study, coordinated the study, and helped draft the manuscript. HH provided critical evaluation on ADP testing and participated in writing of the manuscript. DF conceived the study, performed the statistical analysis, and drafted the manuscript. All authors read and approved the final manuscript.

## Pre-publication history

The pre-publication history for this paper can be accessed here:


